# Child/Adolescent development and autonomous vehicle operation: “operator’s licenses” instead of driver’s licenses

**DOI:** 10.5249/jivr.v10i2.1054

**Published:** 2018-07

**Authors:** David C Schwebel

**Affiliations:** ^*a*^Department of Psychology, University of Alabama at Birmingham, USA.

Autonomous vehicles will be routinely driving on the world’s roadways within the next decade, and they may be ubiquitous by the 2040s.^1-2^Policymakers must heed attention.

There are a range of laws and regulations that might change as a result of autonomous vehicles, but I call attention to one: operator’s licenses. What we today call “driver’s licenses” may soon become outdated artifacts, replaced instead by “operator’s licenses” that document an individual’s capacity to handle any situations that could arise while riding (or sleeping, working, reading, daydreaming, or text-messaging) in an autonomous vehicle driven by a robot.

As the evolution from driving to “operating” occurs over the coming decades, our laws must follow. In particular, what groups of people might comprise safe autonomous vehicle operators who cannot legally drive vehicles? Advocates for the elderly and the disabled will surely lobby government officials, and rightfully so. Another group we must consider is adolescents.

Today’s minimum driving ages were legislated with the cognitive-perceptual limitations of teens in mind. There is evidence to suggest 14-year-olds may not have the capacity to safely operate a vehicle due to the cognitive-perceptual demands required of a driver.^3^ If a robot is driving the vehicle instead, however, might a 14- or 15-year-old safely be transported to and from school without an adult? I tentatively argue the answer is yes. 

Of course, a developmental line will need to be drawn someplace: no 4-year-old could safely ride alone in an autonomous vehicle. Research is needed to investigate the risks and benefits involved, and to offer evidence for law-making. Some American jurisdictions allow children as young as 8 years old to stay home alone and US law permits 14- and 15-year-olds to work in retail and restaurant jobs. Consideration of those and other policies, along with relevant research by developmental scientists, should be taken as we move closer to a world of autonomous vehicles and the need for “operator’s licenses”.
